# Proximal femoral nailing for unstable trochanteric fractures: lateral decubitus position or traction table? A case-control study of 96 patients

**DOI:** 10.1051/sicotj/2024041

**Published:** 2024-11-08

**Authors:** Mohamed I. Abulsoud, Mohamed A.A. Ibrahim, Ahmed Saied Mohammed, Mohammed Elmarghany, Usama Gaber, Elsherbiny Ali Elsherbiny, Samir A. Nematallah, Mohamed Amer Mohamed, Mohamed F. Elhalawany, Yahia A. Hasanien, Mostafa Abonnour

**Affiliations:** 1 Department of Orthopaedic Surgery, Faculty of Medicine, Al-Azhar University Cairo Egypt; 2 Abo-Khalifa trauma and specialized surgeries hospital Ismailia Egypt

**Keywords:** Proximal femoral nail, Intertrochanteric femoral fractures, Patient positioning, Traction table, Harris hip score, Lateral position, Hip fractures

## Abstract

*Purpose*: This study aimed to compare the treatment of unstable intertrochanteric femoral fractures with short proximal femoral nailing in elderly patients in the lateral decubitus position versus the supine position on traction tables. *Methods*: From June 2020 to January 2022, a prospective case-control study was performed on 96 patients who presented with unstable trochanteric fractures treated by internal fixation via short proximal femoral nail (PFN). Patients were divided into two groups: *Group A*, which included patients who underwent surgery in the lateral position; and *Group B*, which included those in the supine position. Both groups were subjected to follow-up for 12 months. *Results*: The mean setup time, surgery time, and blood loss were significantly greater in Group B than in Group A, while the hospital stay and fluoroscopy duration were similar in both groups. Regarding reduction quality and fixation (TAD (tip-apex distance), CDA (collodiaphyseal angle), and Reduction CRQC (change reduction quality criterion)), there were no statistically significant differences between the two groups; moreover, there were no intraoperative or postoperative complications in either group or the Harris hip score (67.65 ± 17.06 in Group A vs. 67.15 ± 17.05 in Group B). *Conclusion*: The lateral decubitus and supine positions on a traction table are suitable for proximal femoral nailing with comparable outcomes, and surgeons can use either position according to their preferences and resources.

## Introduction

Intertrochanteric fractures are among the most frequently managed fractures in orthopaedic practice. The incidence of intertrochanteric fractures is 30.1%. Its incidence is predicted to increase in the coming years owing to population ageing and an increase in the incidence of osteoporosis [1]. Intertrochanteric fractures are the most common type of fracture in elderly patients and have the highest perioperative mortality rate among those with surgically and non-surgically treated fractures [2, 3].

According to their configuration, intertrochanteric fractures are classified as stable or unstable [4]. Unstable trochanteric fractures in elderly individuals are challenging due to associated comorbidities and poor bone quality [5]. The goal of surgical management is to allow early pain-free mobilization to avoid the complication of being bedridden as much as possible [6].

The treatment of such fractures with a dynamic hip screw is associated with a high prevalence of shortcomings, such as unacceptable shortening, external rotation deformity, screw cut out, varus collapse, and secondary surgical procedures, in osteoporotic geriatric patients [7, 8]. Intramedullary fixation has shown superior mechanical advantages, early postoperative ambulation, and faster recovery than plate fixation. Therefore, cephalomedullary femoral nails are currently the implant of choice for treating unstable intertrochanteric fractures. It has the advantages of less risk of fixation failure, less blood loss, and a short hospital stay [9, 10].

Several studies have discussed the advantages and disadvantages of different positioning approaches for performing proximal femoral nailing for trochanteric fractures. The routine method is to perform surgery on a traction table while the patient is in the supine position. However, fixing a patient on a traction table is challenging and time-consuming in addition to the reported complications [11, 12]. In 2010, Ozkan et al. [13] described successful proximal femoral nailing surgery in the lateral decubitus position on a radiolucent table without the need for a traction table.

In 2019, Kakumanu et al. [14] performed proximal femoral nailing in the lateral decubitus position and reported that the lateral position allows easier identification of entry points and a shorter operation time. Lateral positioning also enables conversion to other approaches when needed [15]. However, the lateral decubitus position carries the potential for limited fluoroscopic visualization of the femoral head in the lateral view, especially when proximal locking devices are inserted; therefore, there is no definitive agreement on the best positioning approach when performing proximal femoral nailing for hip fractures, especially in geriatrics.

This study aimed to compare the surgical treatment of unstable intertrochanteric femoral fractures with short proximal femoral nailing in elderly patients in the lateral decubitus position versus the supine position on the orthopaedic table.

## Materials and methods

### General data

From June 2020 to January 2022, a prospective case-control study was undertaken at Al-Azhar University hospitals (Al-Hussein and Bab Elshaerrya hospitals), Cairo, Egypt, on 96 patients who presented with unstable intertrochanteric fracture and were treated with a short proximal femoral nail (PFN). Participants were allocated into two groups based on the day of operation. Patients who were scheduled on Sunday and Monday were subjected to surgery via the lateral decubitus method. Patients who underwent surgery on Tuesday and Wednesday were treated in the supine position on the traction table.

All the patients were evaluated via a routine laboratory investigation and multidisciplinary and anaesthesia consultation before surgery to optimize the patients for surgery, and consent for the operation was obtained.

*The inclusion criteria for patients were as follows*: (1) were adults aged 60 years or older (male and female), (2) had unstable intertrochanteric fractures confirmed with either plain X-rays or computed tomography, (3) had short proximal femoral nails, (4) had recent fractures within 14 days after trauma, (5) were ambulatory before injury, and (6) signed informed consent forms by themselves or their immediate family members.

*The exclusion criteria for patients were as follows*: (1) aged younger than 60 years, (2) had a stable trochanteric fracture, (3) had an old fracture (more than 14 days), (4) had a pathological fracture, (5) had an open fracture, (6) had multiple fractures, and (7) were treated with long nails. (8) Patients with fractures that needed augmentative implants, such as cables or cerclage wires. (9) Patients who did not continue follow-up time (12 months) due to mortality, absence, travelling away…etc.

### Surgical procedure

The institutional Ethics Committee authorized this investigation, and all study participants gave both written and verbal informed consent from themselves and their first-degree relatives. Low-molecular-weight heparin was used for antithrombotic prophylaxis in all patients beginning at the time of hospitalization, was discontinued 12 h before surgery, and was reinitiated for 1 month in both groups. The same device and implant material (titanium short proximal femoral nail, Orthomed^®^, Egypt) were used for all patients. In all the groups, nails were inserted using the same normal process.

With the introduction of anaesthesia, all patients received prophylactic antibiotics (2 gm. third-generation cephalosporins) and were administered under spinal or epidural anaesthesia.

*In the lateral decubitus position*, the patients were positioned on a radiolucent table. Pelvic stabilization is achieved by positioning the pelvic post. All bony prominences were well protected. The affected extremity is positioned up with the hip extended, and the other limb is positioned with flexion of the hip to accommodate fluoroscopic visualization with the use of a C-arm.

**Figure 1:**
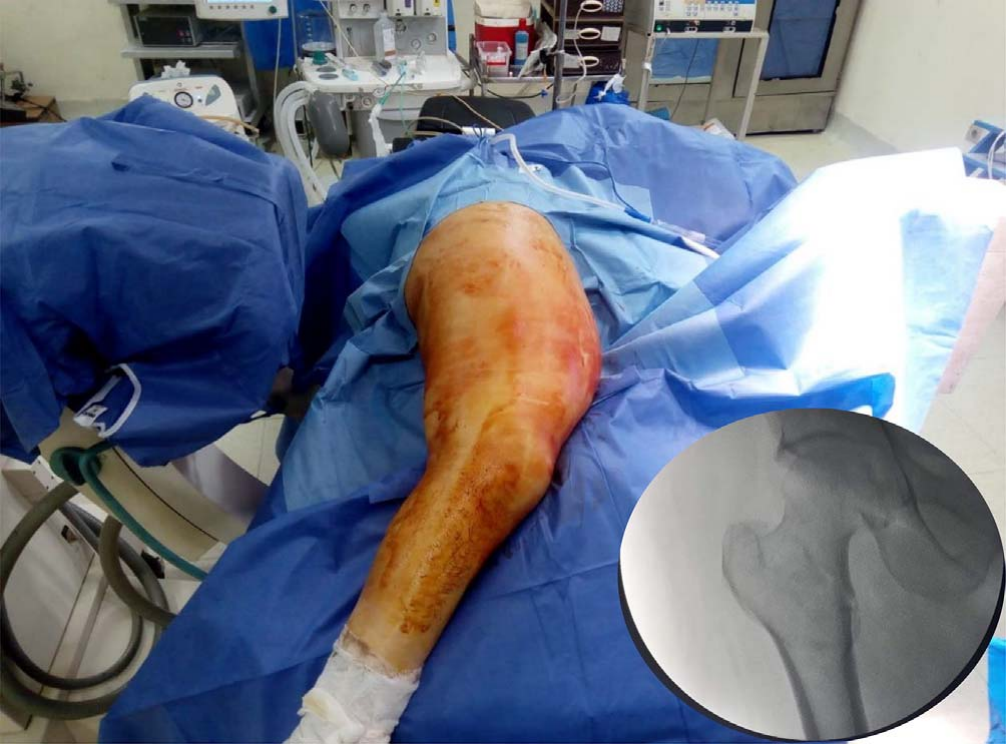
The position of the patient in the lateral decubitus position after draping the patient and the C-arm. The bottom left photo shows the obtained X-ray without traction.

An acceptable reduction can be achieved by gentle traction, internal rotation, and adduction. In this position, the weight of the limb is sufficient to keep the reduction stable (Figure 1).

The challenge with this technique is obtaining a clear lateral view. While an anteroposterior (AP) X-ray can easily be obtained by rotating the c arm under the table, the use of “frog leg” imaging for a lateral X-ray is not recommended because it leads to a loss of reduction and cannot provide clear lateral imaging.

**Figure 2:**
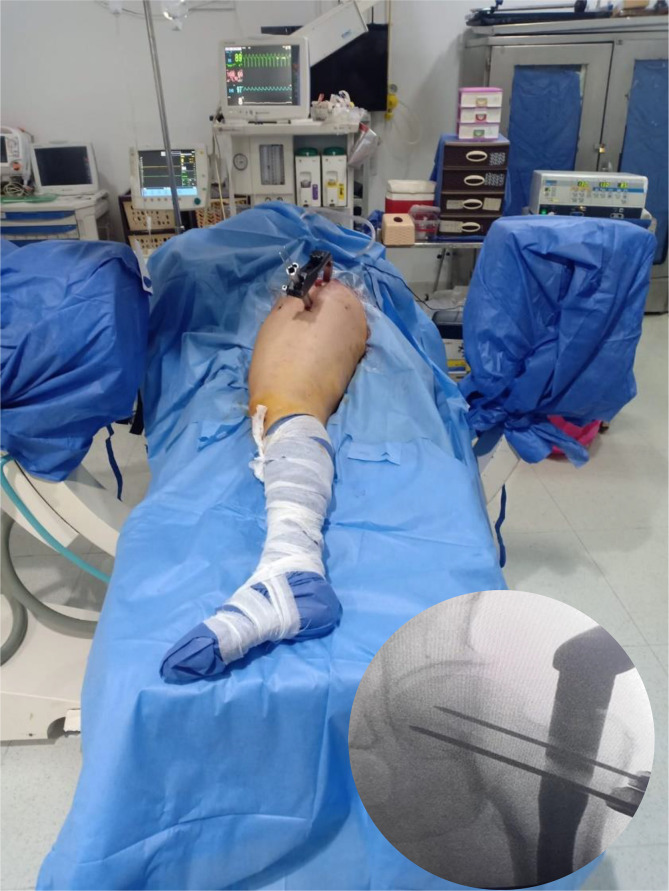
The position of the lower limb, and the bottom fluoroscopic image shows the position of the guidewires for proximal fixation after the insertion of the nail.

**Figure 3:**
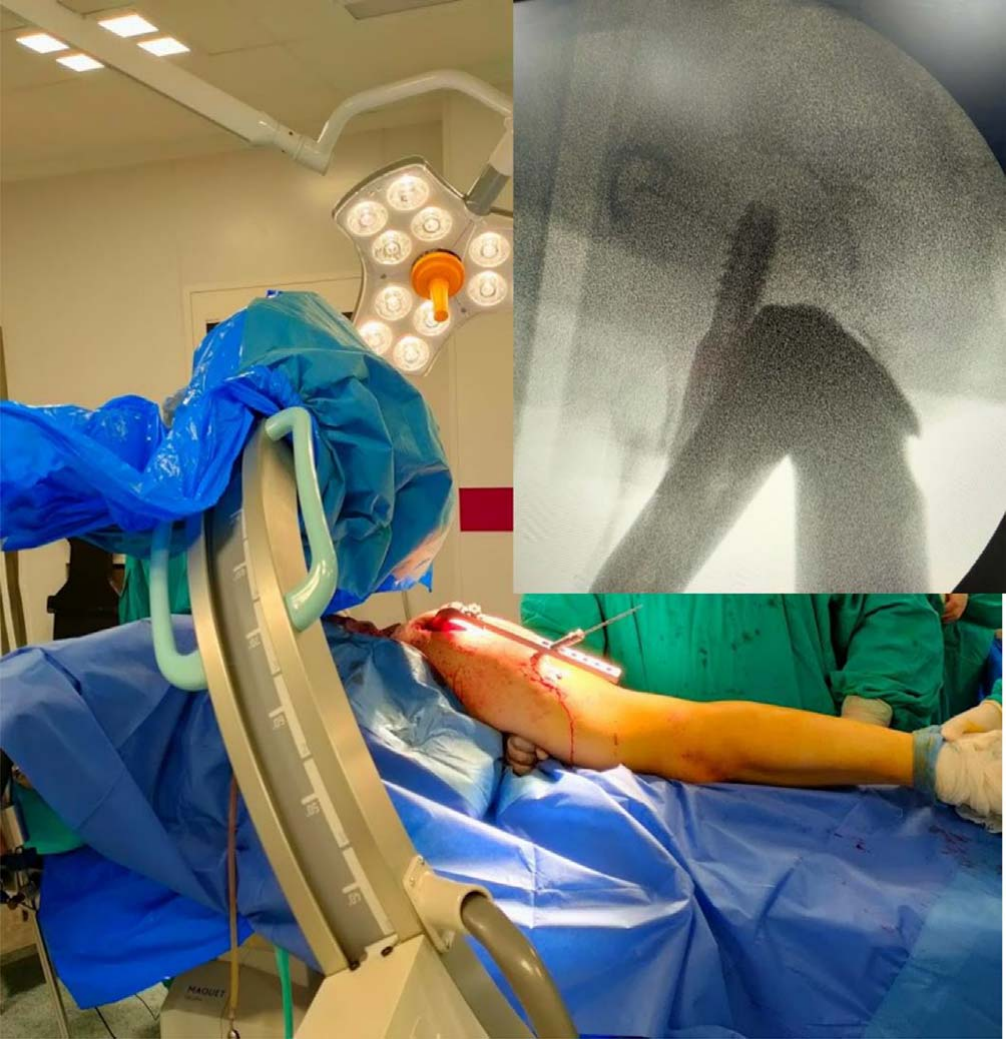
The method of obtaining a lateral view in the presence of the metallic inserter by tilting the fluoroscope 30–45° cephalic, and the top left fluoroscopic image shows the image obtained using this manoeuvre.

**Figure 4:**
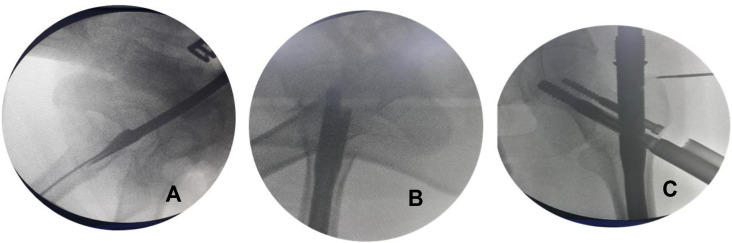
Fluoroscopic images showing the technique of nailing for unstable trochanteric fractures in the lateral decubitus position without a traction table. (A) The entry point when the guidewire was passed through the lateral position. (B) The final image after inserting the proximal screws in the neck of the femur in the lateral view. (C) The position of the proximal screws in an anteroposterior view.

A lateral view could be obtained by turning the tube of the C-arm 90° on the radiolucent table. The beams are directed 30–40° degrees caudally centred on the greater trochanter. Using this method, the operated femoral head and neck can be observed in the lateral view even in the presence of the metallic jig inserter (Figures 2–4).

A longitudinal incision started 4–5 cm proximal to the tip of the greater trochanter. The incision was made in the fascia lata, which was split in line with its fibres. The point of entry at the tip of the greater trochanter at its centre was confirmed by the C-arm in both AP and lateral views. The guide wire was inserted in the centre of the medullary canal. The 17.0 mm reamer is used first for the proximal femur through the protection sleeve over the guide wire and renamed manually with the universal chuck with a T-handle as far as the stop on the protection sleeve. Reaming was performed with a 0.5 mm increment in the distal fragment to fit thicker nails, and the proper nail was manually inserted. The nail was inserted completely into the femur until the proximal tip was flush with the tip of the greater trochanter.

**Figure 5:**
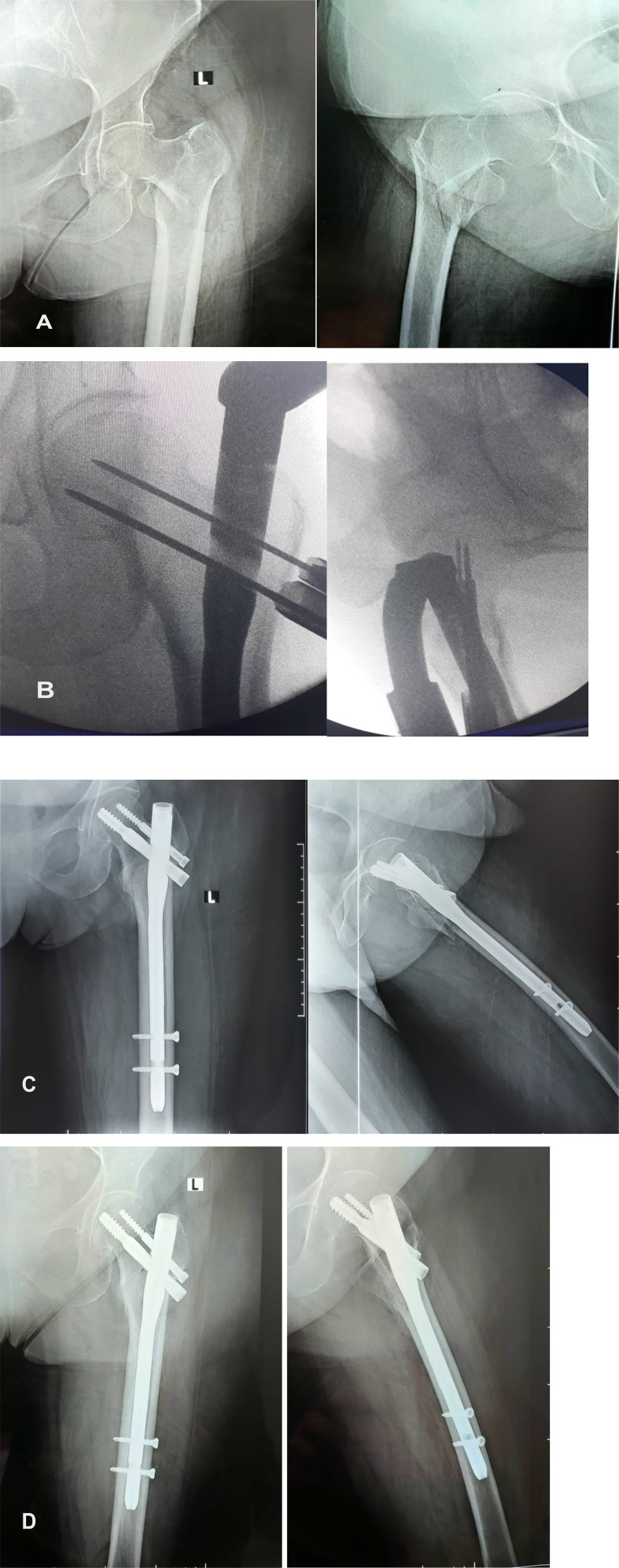
The case of a patient with an intertrochanteric femoral fracture managed by proximal femoral nailing in the lateral decubitus position without a traction table. (A) Preoperative X-ray of a 73-year-old woman who presented with an unstable trochanteric fracture. (B) Intraoperative fluoroscopic photos showing the position of the guidewires for the proximal locking screws (lag screw and antirational screw). (C) Postoperative X-ray of the proximal femur showing the position of the nail and the central position of the lag screw. (D) Follow-up X-ray image showing healing of the fracture with a stable implant position.

Visualization of guide wires for the proximal locking screws in the femoral neck and head on the C-arm obtained through AP and lateral views as described earlier. The placement of the guide wire should be central to the femoral head. The rotational alignment of the fracture was assessed by checking the position of the patella, which should be perpendicular to the floor in a neutral position. Drilling was performed over the guide wire with 6.4 mm and 8 mm drill bits to the desired length and confirmed by a C-arm. A distal femoral neck self-tapping screw of 11 mm was inserted, and a proximal anti-rotation self-tapping screw of 6.5 mm was inserted. The proximal screw is typically 10–15 mm shorter than the femoral neck screw. Distal locking was performed with self-tapping 4.9 mm bolts. After fixation was complete, thorough lavage was performed with normal saline. Hemostasis was achieved, and the incision was closed in layers. A sterile dressing was applied over the wound (Figure 5).

**Figure 6:**
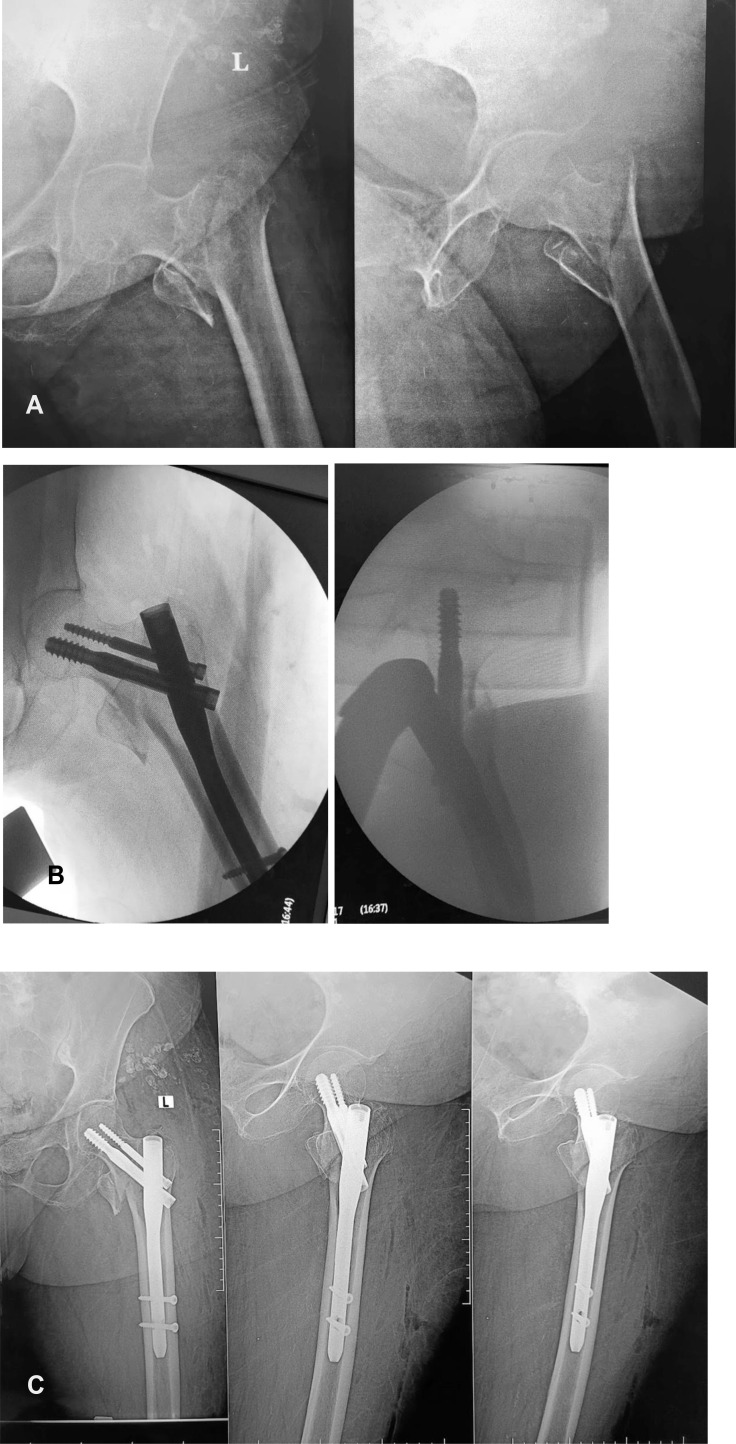
The case of a patient with an intertrochanteric femoral fracture managed by proximal femoral nailing using a traction table in the supine position. (A) Preoperative X-ray of a 71-year-old woman who presented with an unstable trochanteric fracture. (B) Intraoperative fluoroscopic photos showing the position of the proximal locking screws (lag screw and antirational screw). (C) Postoperative X-ray image of the proximal femur showing the position of the nail and the central position of the lag screw, the TAD is less than 25 mm, CDA is about 135°, and position of proximal locking screws according to the Cleveland and Bosworth quadrants in quadrant number 5, and the quality of reduction was good according to the CRQC.

*In the other group, in the supine position on the traction table*, the fractured limb was placed in the boot traction position, and the contralateral limb was placed in the abduction or hemi-lithotomy position to allow proper lateral imaging of the affected hip. The traction was applied, the fracture was reduced with traction and internal rotation, and the reduction in the adduction manoeuvre was checked under fluoroscopy on the AP and lateral views. The operation was completed following standard PFN procedures as prescribed in the lateral position group (Figure 6).

### Measurement parameters

The setup (preparation) time in the operating room which is the time between the end of anaesthesia and the time-out in both groups was recorded. The actual operation time was recorded from the start of the surgical incision until skin closure in both groups. The total anaesthesia time (setup time + surgical time), fluoroscopy time, bleeding volume (the number of gauze pads [× 10 ccs] and amount in the aspirator jar were recorded. After surgery, AP and lateral X-rays on the operated hip were taken and reviewed by the authors. In both groups, the tip-apex distance (TAD) (<25 mm) [16], collodiaphyseal angle (CDA) (∼135°) [17], and position of proximal locking screws according to the Cleveland and Bosworth quadrants [18] were recorded for comparison, and the quality of reduction was determined using the change reduction quality criterion (CRQC) [19].

#### Postoperative follow-up protocol and data collection

Antibiotics, anticoagulants, and pain control agents were administered according to the hospital’s instructions after the patient was admitted to the hospital. Sterile dressing was applied to the wound 48 h after surgery, and the epidural catheter was kept for postoperative analgesia. Sutures were removed between 15 and 21 days after surgery. Static exercises and mobilization were initiated on the first postoperative day. All patients were followed up at an interval of 3–4 weeks until fracture union and then once every 3 months until 1 year. Patients were either allowed partial weight-bearing as tolerated using a frame walker or non-weight-bearing in the first 6 weeks based on intraoperatively achieved stability and postoperative radiographic findings. At each visit, X-rays were taken until fracture union, at which time the patients were evaluated clinically for hip and knee function, walking capacity, fracture union, deformity, and shortening. In addition, at 3-, 6-, 9-, and 12-months post-surgery. All complications were documented through the last follow-up. The functional outcome of surgery was determined based on the Harris hip score (HHS), which has been validated for the measurement of the clinical outcome of proximal femoral fractures in elderly patients. HHS is a score of 100 points (the higher the score is, the better the function is), which consists of 4 items (pain 44 points, function 47 points, absence of deformity 4 points, and range of motion (ROM) 5 points). The questionnaire consists of eight questions: one for pain and seven for function, and two observer measures: one for ROM and one for the absence of deformity.

### Statistical analysis

The Statistical Package for the Social Sciences (SPSS) (v 25, Armonk, NY, USA) was used to analyze the data. The quantitative data are presented as the mean and range, while the qualitative data are presented as the frequency and percentage. Comparisons between variables before and after surgery were made using the Mann-Whitney *U* test. *P* values less than 0.05 were considered to indicate statistical significance.

## Results

The study included 96 patients who presented with unstable trochanteric femoral fractures. Patients were allocated into two groups: Group A, which included 48 patients who had undergone surgery in the lateral decubitus position; and Group B, which included 48 patients who had undergone surgery using the traction table in the supine position.

Concerning demographic data, there was no statistically significant difference between the two groups regarding age, sex, fracture type, or comorbidities (Table 1).

**Table 1:** Demographic data.

Variable	Group A: Lateral decubitus position (*n* = 48)	Group B: Supine position (*n* = 48)	P^(B)^
Age	71.81 ± 6.31	71.47 ± 5.74	0.77
Gender			
Male (*n* = 45)	24 (50.0%)	21 (43.8%)	0.68
Female (*n* = 51)	24 (50.0%)	27 (56.3%)	
Side			
Right (*n* = 47)	26 (54.2%)	21 (43.8%)	0.41
Left (*n* = 49)	22 (45.8%)	27 (56.3%)	
Type/AO			
A2 (*n* = 63)	32 (66.7%)	31 (64.6%)	1.00
A3 (*n* = 33)	16 (33.3%)	17 (35.4%)	
Diabetes miletus	28 (58.3%)	30 (62.5%)	0.68
Hypertension	32 (66.7%)	29 (60.4%)	0.51
Ischemic heart disease	24 (50%)	21 (43.8%)	0.54

The mean setup time (15.54 ± 1.62 minutes in group A, 17.71 ± 1.66 min in group B), surgery time (53.96 ± 4.19 minutes in group A, 67.27 ± 6.28 min in group B), and blood loss volume (151.88 ± 10.75 mL in group A, 191.46 ± 10.16 mL in group B), were significantly greater in Group B than in Group A (*P*-value < 0.001), while the hospital stay and fluoroscopy duration were similar between the two groups (Table 2).

**Table 2:** The results of the intraoperative variables in both groups.

Variable^(A)^	Group A: Lateral decubitus position (*n* = 48)	Group B: Supine position (*n* = 48)	*P* ^(B)^
Hospital stay (days)	12.25 ± 1.36	12.06 ± 1.26	0.48
Set-up time (min)	15.54 ± 1.62	17.71 ± 1.66	<0.001*
Operative time (min)	53.96 ± 4.19	67.27 ± 6.28	<0.001*
Blood loss (mL)	151.88 ± 10.75	191.46 ± 10.16	<0.001*
X-ray exposure (s)	48.38 ± 5.53	50.88 ± 7.07	0.06

P value ≤ 0.05 is considered significant (*).

Concerning the quality of reduction and fixation (TAD, CDA, and Reduction CRQC), there were no statistically significant differences between the two groups (Table 3).

**Table 3:** Results of the quality of reduction and fixation in both groups.

Variable	Group A: Lateral decubitus position (*n* = 48)	Group B: Supine position (*n* = 48)	*P*
TAD –Tip apex distance (mm)	24.81 ± 2.18	25.88 ± 3.23	0.06
CDA – Collodiaphyseal angle (°)	132.17 ± 5.10	130.71 ± 5.50	0.18
Reduction CRQC	3.02 ± 0.96	3.25 ± 0.93	0.24
Good: 4 (*n* = 42)	18 (37.5%)	24 (50.0%)	0.48
Acceptable: 2, 3 (*n* = 46)	26 (54.2%)	20 (41.7%)	
Poor: 0, 1 (*n* = 8)	4 (8.3%)	4 (8.3%)	

There were no statistically significant differences in the incidence of intraoperative or postoperative complications in either group or the HHS (67.65 ± 17.06 in Group A vs. 67.15 ± 17.05 in Group B) (Table 4).

**Table 4:** The incidence of intraoperative or postoperative complications in both groups.

Variable^(A)^	Group A: Lateral decubitus position (*n* = 48)	Group B: Supine position (*n* = 48)	*P* ^(B)^
Anterior cortex fracture	0 (0.0%)	0 (0.0%)	–
Fracture displacement by nail insertion	6 (12.5%)	8 (16.7%)	0.77
Failure to get closed reduction	2 (4.2%)	2 (4.2%)	1.00
Varus angulation	7 (14.6%)	6 (12.5%)	1.00
Failure of distal locking	3 (6.3%)	2 (4.2%)	1.00
Superficial wound infection	5 (10.4%)	3 (6.3%)	0.71
Deep wound infection	2 (4.2%)	3 (6.3%)	1.00
Delayed union (>6 months)	7 (14.6%)	3 (6.3%)	0.32
Nonunion	7 (14.6%)	6 (12.5%)	1.00
Malunion	1 (2.1%)	1 (2.1%)	1.00
Implant failure	7 (14.6%)	6 (12.5%)	0.871
Deep venous thrombosis	5 (10.4%)	5 (10.4%)	1.00
Harris hip score (HHS)	67.65 ± 17.06	67.15 ± 17.05	0.89
Follow-up time (months)	15.46 ± 5.64	15.17 ± 5.30	0.79

## Discussion

The study showed that the failure of unstable trochanteric femoral fractures either in the lateral decubitus position or on a supine traction table led to comparable results regarding the quality of reduction and fixation, the incidence of intraoperative or postoperative complications, and the functional outcome according to the HHS 12 months after surgery; however, the setting time, operative time, and blood loss were greater in the traction table group.

The technique of nailing the lateral decubitus position in trochanteric femoral fractures has been described in detail concerning the use of a reproducible method for obtaining a good lateral view while inserting proximal screws even in the presence of metallic insertion devices, which often represents a problem for surgeons who resort to the use of traction tables [13, 14].

The traction table carries the risk of several complications [20]. Perineal complications have recently been reported to be approximately 3% [21]. Additionally, the increased setup time and subsequent surgery time in such typically geriatric populations, without a clear advantage regarding the quality of reduction and fixation, could encourage surgeons, especially those in hospitals where a traction table is not available, to perform such surgery in the lateral position.

Recently, the double reverse traction repositor has been introduced as an option to maintain a reduction in the supine position without the use of a traction table; such a device could be helpful in some cases and clinical situations; however, the inherent invasiveness of the device compared to the lateral decubitus position invites further studies to compare both positioning techniques [22–24].

The quality of reduction is the most important controllable predictor of a good outcome, even patient survival, in proximal femoral fractures [25, 26]; therefore, achieving and maintaining proper reduction during the whole surgery is highly crucial regardless of the positioning technique. Lateral decubitus position nailing has shown comparable results concerning the quality of reduction and fixation in comparison with the traction table. These results have also been reported by Sönmez et al. [27] and Doğan et al. [28].

In the study of Sönmez et al. [27], 82 patients were included. There was no difference between the two groups regarding the quality of reduction, but the surgical time, setting time, and fluoroscopy exposure were greater in the traction table group. The same results were obtained by Doğan et al. [28], who included 80 patients. Comparable results were reported by Souza et al. [29] and Li et al. [30].

The implant used in this study was short PFN, and the implant choice (PFN or PFNA) did not seem to change the results in similar studies [27–30]. Proximal femoral nails give comparable results to those of other intramedullary devices [31, 32] used for a relatively long time [33]; however, the use of metallic jigs was overcome in this study by moving the beam of the fluoroscopy away from it while the patient was in the lateral decubitus position. Moreover, these studies investigated implant failure in both groups, which is similar between both groups; these points have not been covered in previous studies.

The lateral decubitus position has several advantages over the prone position. First, the entry point and the greater trochanter can easily be obtained, especially in obese people, because excessive soft tissue displaces away from the starting point due to gravitational force. Furthermore, accurate fracture reduction and femoral rotation can be achieved by neutralizing the muscle forces causing flexion and abduction of the proximal fracture fragment due to iliopsoas muscle force by increasing flexion of the hip and manual traction, which is easy in the lateral decubitus position.

The study has limitations, it was a single-centre and nonrandomized study because blinding was impossible at our institution. A larger sample size and a multicenter study are needed to verify these results. The lateral decubitus position has a learning curve, and familiarity with this position is needed, especially for surgeons who are more familiar with the traction table.

## Conclusion

The lateral decubitus position on a radiolucent table and the supine position on a traction table are suitable options for applying the proximal femoral nail with similar radiological parameters and functional outcomes. The lateral decubitus position provides a shorter operation time, shorter setting time, and less blood loss than the supine position.

## Data Availability

The datasets used and analyzed during this investigation are accessible upon request from the corresponding author.
